# Perioperative risk of pancreatic head resection—nomogram-based prediction of severe postoperative complications as a decisional aid for clinical practice

**DOI:** 10.1007/s00423-021-02426-z

**Published:** 2022-03-23

**Authors:** J. Hipp, L. Rist, S. Chikhladze, D. A. Ruess, S. Fichtner-Feigl, U. A. Wittel

**Affiliations:** Center of Surgery, Department of General and Visceral Surgery, Medical Center-University of Freiburg, Faculty of Medicine, Hugstetter Str. 55, 79106 Freiburg, Germany

**Keywords:** Nomogram, Perioperative risk, Pancreatic head resection, Pancreatic cancer, IPMN

## Abstract

**Purpose:**

To develop nomograms for pre- and early-postoperative risk assessment of patients undergoing pancreatic head resection.

**Methods:**

Clinical data from 956 patients were collected in a prospectively maintained database. A test (*n* = 772) and a validation cohort (*n* = 184) were randomly generated. Uni- and multi-variate analysis and nomogram construction were performed to predict severe postoperative complications (Clavien-Dindo Grades III–V) in the test cohort. External validation was performed with the validation cohort.

**Results:**

We identified ASA score, indication for surgery, body mass index (BMI), preoperative white blood cell (WBC) count, and preoperative alkaline phosphatase as preoperative factors associated with an increased perioperative risk for complications. Additionally to ASA score, BMI, indication for surgery, and the preoperative alkaline phosphatase, the following postoperative parameters were identified as risk factors in the early postoperative setting: the need for intraoperative blood transfusion, operation time, maximum WBC on postoperative day (POD) 1–3, and maximum serum amylase on POD 1–3. Two nomograms were developed on the basis of these risk factors and showed accurate risk estimation for severe postoperative complications (ROC-AUC-values for Grades III–V—preoperative nomogram: 0.673 (95%, CI: 0.626–0.721); postoperative nomogram: 0.734 (95%, CI: 0.691-0.778); each *p* ≤ 0.001). Validation yielded ROC-AUC-values for Grades III–V—preoperative nomogram of 0.676 (95%, CI: 0.586–0.766) and postoperative nomogram of 0.677 (95%, CI: 0.591–0.762); each *p* = 0.001.

**Conclusion:**

Easy-to-use nomograms for risk estimation in the pre- and early-postoperative setting were developed. Accurate risk estimation can support the decisional process, especially for IPMN-patients with an increased perioperative risk.

**Supplementary Information:**

The online version contains supplementary material available at 10.1007/s00423-021-02426-z.

## Introduction

Despite ongoing progress in surgical technique and perioperative management of patients, pancreatic head resection remains a challenging procedure with a substantial rate of perioperative complications and perioperative mortality. While surgical resection of pancreatic ductal adenocarcinoma (PDAC) and other malignant tumors of the pancreas is inevitable and in most cases the patient’s only chance for cure, the decision for surgical resection due to benign and premalignant lesions has to be weighed between the risk of malignant transformation on the one hand and surgical risk and postoperative quality of life on the other hand [1]. Especially in patients with intraductal papillary mucinous neoplasms (IPMN) there is a risk of overtreatment for patients with no risk factors and worrisome features from an oncological perspective [2] and—simultaneously—these patients are at increased risk of postoperative complications after pancreatic head resection [3]. A well-considered recommendation has to be offered to a patient by the treating physicians in this situation.

A nomogram is a simple tool which creates a graphic representation of a statistical predictive model [4]. Risk scores and nomograms can help surgeons assess the individual risk of a patient’s procedure and can help to guide the decision-making process with a patient to achieve informed consent as well [5].

While a variety of risk scores for prediction of oncologic survival after resection of PDAC exist [6–11], predictive risk scores or nomograms for summarized postoperative surgical outcome are lacking besides specialized models for the prediction of pancreatic fistula [12, 13], intraabdominal infectious complications after pancreatic resection [14], and the risk of postoperative mortality after pancreatic surgery [15, 16].

Therefore, the first aim of our study was the development of a simple-to-use nomogram to preoperatively estimate the perioperative risk of severe complications following pancreatic head resection to support the preoperative decisional process. The second aim of our study was the development of a postoperative nomogram to predict the probability of severe complications in the postoperative course on the basis of information available at the end of the third postoperative day.

## Methods

### Patient selection

Patient data from 956 consecutive pancreatic head resections for several indications were collected in a prospectively-maintained database and evaluated retrospectively. Operations were performed between August 2001 and October 2018. The study was reviewed and approved by the Ethics Committee of the University of Freiburg (EK 85/20).

### Data management

According to the study protocol, the following clinical and laboratory data were extracted from the prospectively-maintained database: demographic data (age, gender), clinical data (indication for surgery, ASA score [17], body mass index (BMI), performed operation including reconstruction, intraoperative blood loss, intraoperative blood transfusions, operation time, texture of pancreatic tissue, postoperative complications, diagnostics of complications, and treatment of complications and all available laboratory data of routine laboratory monitoring. Complications were classified according to the Clavien-Dindo general classification of complications [18] and the comprehensive complication index (CCI) [19]. Pancreatic surgery-specific complications such as postoperative pancreatic fistula (POPF) [20], delayed gastric emptying (DGE) [21], and post-pancreatectomy hemorrhage (PPH) [22] were classified according to International Study Group of Pancreatic Surgery (ISGPS) definitions.

All available results of the routine perioperative laboratory monitoring were collected from the preoperative laboratory evaluation to postoperative day (POD) 14. Laboratory samples were categorized according to the postoperative time, beginning from the preoperative sample (POD 1) to the postoperative samples on POD 1 to 3 (POD 1–3), POD 4 to 6 (POD 4–6), POD 7 to 9 (POD 7–9), and POD 10 to 14 (POD 10–14). For each time interval, the maximum observed value of a certain parameter was chosen as the value for the corresponding time interval. The following parameters of routine laboratory monitoring from blood-samples were collected in the study database: white blood cell (WBC) count, C-reactive protein (CRP), procalcitonin (PCT), serum amylase (amylase), serum lipase (lipase), aspartate aminotransferase/glutamate oxaloacetate transaminase (AST/GOT), alanine aminotransferase/glutamate pyruvate transaminase (ALT/GPT), gamma-glutamyltransferase (GGT), alkaline phosphatase (AP), serum total bilirubin (bilirubin), creatinine and blood urea nitrogen (BUN), the preoperative carbohydrate antigen 19-9 (CA19-9) value, the preoperative International Normalized Ratio (INR) value, and the preoperative partial thromboplastin time (PTT). Drainage samples were evaluated for drain amylase on the first three postoperative days. Acute kidney injury on POD 1–3 was diagnosed when creatinine increased 1.5 times over the preoperative value according to RIFLE-criteria [23].

### Statistical analysis

Patients were randomly assigned to two groups of about 80% and 20% of the entire cohort. A total of 772 patients (80.8%) were assigned to the test cohort and 184 (19.2%) were assigned to the validation cohort. Results are expressed as median ± interquartile range (IQR) or as number (percent). Categorical variables were compared using Pearson’s chi-square test. Mann-Whitney *U*-test or Kruskal-Wallis test with Mann-Whitney *U*-post hoc test and Bonferroni correction were used for descriptive analysis of non-parametric variables.

As the aim of this study was the development of a diagnostic screening tool to select patients at risk for postoperative complications after pancreatic head resection, patients of the test cohort were grouped according to Clavien-Dindo classification Grades 0–II and III–V. Univariate analysis was performed using Pearson’s chi-square test or Mann-Whitney *U*-test as applicable. Variables with a *p*-value < 0.1 were included in multivariate analysis. In addition to this variable selection, only one variable of possibly confounding variables (e.g., only one variable for each organ system) was selected for multivariate analysis and—in respect of the study aim—only preoperatively-known parameters were included in the multivariate analysis for the preoperative nomogram and only parameters with a known outcome on POD 3 were included in the variable selection for the multivariate analysis of the postoperative nomogram. For multivariate analysis and nomogram development, continuous variables were dichotomized by the optimal cutoff-value according to the results of the receiver operating characteristics (ROC) analysis with Youden index calculation [24]. Cutoff-values below laboratory threshold values were set to the laboratory threshold value (GPT POD 1 and AP POD 1). Multivariate analysis was then performed by binary logistic regression with backward stepwise variable selection. Nomograms were constructed based on the developed multivariate models [25]. Internal validation in the test cohort was performed by bootstrap resampling with 100 repetitions. External validation was performed with the validation cohort and the area under the curve (AUC) of the ROC analysis. The fistula risk score (FRS) was calculated as previously reported by Callery et al. [26]. Statistical analysis was performed using SPSS version 24.0.0.1 (IBM Corp., Armonk, NY, USA) and R version 4.0.0 (R Foundation for Statistical Computing, Vienna, Austria) with RStudio (RStudio Inc., Boston, MA, USA) and additional packages *rms,* *ggplot*, and *pROC*. Differences were considered statistically significant at *p* < 0.05.

## Results

### Clinical and routine laboratory characteristics of test cohort

The clinicolaboratory characteristics of the test cohort are summarized in Tables 1 and 2 and of the validation cohort in “Supplementary information” Table S1. During the perioperative course, several parameters of routine laboratory monitoring of the patients showed statistically significant differences between patients with and without severe postoperative complications. WBC showed an early postoperative peak on POD 1–3—as expected—with decreasing values in both groups of patients on POD 4–6 followed by a second increase in both groups on POD 7–9. While the patients without severe complications also showed an increase in WBC on POD 7–9, patients with severe complications showed persisting leukocytosis on POD 10–14 and significantly elevated WBC during the entire postoperative course (Fig. 1A). CRP showed higher elevation on POD 1–3 in patients with severe complications and persisting CRP-values > 100 mg/l after POD 10 in contrast to adequately decreasing CRP-values in patients without severe complications (Fig. 1B). PCT showed elevated values in patients with severe complications as well, but was excluded from further statistical evaluation due to the low number of performed tests and a high degree of patient selection. Serum amylase showed a peak on POD 1–3 in patients with severe complications, indicating postoperative pancreatitis as a sign for developing POPF while serum amylase-values below clinical threshold value on POD 1–3 were associated with an uneventful postoperative course. Interestingly, although serum amylase normalized below clinical cutoff-value in both groups, high-normal amylase values (≥ 7 U/l) were seen in the group with postoperative complications while significantly lower-normal values were observed in those patients without complications (Fig. 1C). Serum lipase values showed similar variations as the serum-amylase during the postoperative period. A significant difference between the patients with and without severe postoperative complications could be observed for the renal retention parameters creatinine and BUN for the entire perioperative time, indicating that chronic and acute renal failure are associated with perioperative risk of complications. The GOT showed an early peak on POD 1–3 in patients with complications, indicating its usefulness in diagnostics of postoperative liver perfusion complications. Bilirubin showed no differences in the early postoperative course but patients with complications showed elevated bilirubin after POD 7. An interesting observation was made concerning alkaline phosphatase. In our study cohort, higher preoperative values are associated with lower risk for postoperative complications. A statistically significant association between the texture of the pancreatic tissue and the preoperative value of alkaline phosphatase could be detected (*p* < 0.001). Correspondingly, a significant difference between the POPF and no POPF-groups was observed as well (*p* < 0.001, Kruskal-Wallis ANOVA with Mann-Whitney *U*-post hoc test, Supplementary information Table S2).

**Table 1:** Clinical data of test cohort

	Clavien-Dindo Grades 0–II (*n* = 565)	Clavien-Dindo ≥ Grade III (*n* = 207)	*p*-value
Age (years) (*n* = 772)	66 (55**–**74)	67 (59**–**74)	0.045#
Gender			0.612*
Male	316 (55.9%)	120 (58.0%)	
Female	249 (44.1%)	87 (42.0%)	
Disease			0.001*
PDAC	324 (57.3%)	97 (47.1%)	
Distal bile duct cancer	35 (6.2%)	25 (12.1%)	
Duodenal carcinoma	12 (2.1%)	12 (5.8%)	
Metastases	6 (1.1%)	1 (0.5%)	
pNET	28 (5.0%)	7 (3.4%)	
IPMN	37 (6.5%)	23 (11.2%)	
Chronic pancreatitis	86 (15.2%)	23 (11.2%)	
Other	37 (6.5%)	18 (8.7%)	
Indication (risk-stratified)			< 0.001*
Low-risk indication (PDAC, CP, pNET, metastases)	444 (78.6%)	128 (62.1%)	
High-risk indication (IPMN, bile duct cancer, duodenal cancer, other indications)	121 (24.4%)	78 (37.9%)	
ASA score			0.001*
ASA 1–2	313 (62.2%)	93 (48.4%)	
ASA 3–4	190 (37.8%)	99 (51.6%)	
BMI			0.023*
BMI < 30 Kg/m^2^	515 (91.2%)	177 (85.5%)	
BMI ≥ 30Kg/m^2^	50 (8.8%)	30 (14.5%)	
Preoperative CA19-9 (U/ml) (*n *= 487)	42.6 (14.5**–**256.15)	28.5 (10**–**116)	0.032#
Stent/PTCD preoperative			0.211*
No	291 (52.2%)	117 (57.4%)	
Yes	266 (47.8%)	87 (42.6%)	
Operation			0.573*
Whipple procedure	59 (10.4%)	26 (12.6%)	
PPPD	382 (67.6%)	141 (68.1%)	
Lap. ass. PPPD	124 (21.9%)	40 (19.3%)	
Portal vein resection			0.150*
No	429 (76.1%)	166 (81.0%)	
Yes	135 (23.9%)	39 (19.0%)	
Multivisceral resection			0.971*
No	507 (89.9%)	185 (89.8%)	
Yes	57 (10.1%)	21 (10.2%)	
Neoadjuvant treatment (PDAC patients)			0.818*
No	299 (95.2%)	91 (95.8%)	
Yes	15 (4.8%)	4 (4.2%)	
Adjuvant treatment (PDAC patients)			0.001*
No	43 (16.0%)	26 (32.5%)	
Yes	225 (84.0%)	54 (67.5%)	
Operation time (min.) (* n *= 771)	418 (352**–**475)	440 (376**–**522)	< 0.001#
Intraoperative blood loss (ml) (*n *= 453)	600 (400**–**950)	600 (400**–**1 175)	0.409#
Intraoperative blood transfusion			< 0.001*
No	492 (87.1%)	153 (73.9%)	
Yes	73 (12.9%)	54 (26.1%)	
Pancreatic tissue			0.001*
Hard texture	123 (47.3%)	26 (28.3%)	
Soft texture	137 (52.7%)	66 (71.7%)	
Reconstruction			0.623*
Pancreatogastrostomy	224 (39.7%)	89 (43.2%)	
Pancreaticojejunostomy	336 (59.6%)	115 (55.8%)	
No reconstruction	4 (0.7%)	2 (1.0%)	
POPF ISGPS			< 0.001*
Grade B	68 (12.0%)	44 (21.7%)	
Grade C	0 (0%)	52 (25.6%)	
Days with easy flow drain (*n *= 715)	6 (5**–**10)	12 (6**–**30)	< 0.001#
Maximum drain amylase POD 1-3 (U/l) (*n* = 287)	3 344 (575**–**14 869)	6 385 (1 775**–**27 039)	0.004#
Days with octreotide (*n* = 752)	0 (0**–**6)	4 (0**–**7)	< 0.001#
DGE ISGPS			< 0.001*
Grade B	55 (9.7%)	33 (13.3%)	
Grade C	7 (1.2%)	66 (32.5%)	
PPH ISGPS			< 0.001*
Grade B	13 (2.3%)	29 (14.2%)	
Grade C	0 (0%)	35 (17.2%)	
Clavien-Dindo classification			< 0.001*
Grade 0-II	565 (100%)	0 (0%)	
Grade III	0 (0%)	157 (75.8%)	
Grade IV	0 (0%)	21 (10.1%)	
Grade V	0 (0%)	29 (14.0%)	
Comprehensive complication index (*n* = 772)	20.90 (0**–**22.60)	44.90 (38.10**–**63.00)	< 0.001#
Postoperative hospital stay (days) (*n *= 760)	14 (12**–**18)	30 (21**–**43)	< 0.001#

*Pearson’s chi-square test

#Mann-Whitney *U*-test

**Table 2:** Perioperative course of routine laboratory parameters of test cohort

	Clavien-Dindo Grades 0–II (*n* = 565)	Clavien-Dindo ≥ Grade III (*n* = 207)	*p*-value
WBC POD 1 (x10^3^/μl) (*n* = 751)	7.06 (5.76**–**8.60)	7.20 (6.05**–**9.11)	0.077#
WBC POD 1–3 (x10^3^/μl) (*n* = 764)	13.21 (10.80**–**15.97)	14.40 (11.46**–**17.24)	0.004#
WBC POD 4–6 (x10^3^/μl) (*n* = 746)	9.20 (7.24**–**11.58)	11.50 (9.19**–**14.70)	< 0.001#
WBC POD 7–9 (x10^3^/μl) (*n* = 691)	10.17 (8.22**–**12.83)	15.60 (11.96**–**21.43)	< 0.001#
WBC POD 10–14 (x10^3^/μl) (*n* = 650)	10.70 (8.10**–**13.71)	17.07 (12.89**–**23.19)	< 0.001#
CRP POD 1 (mg/l) (*n* = 298)	8 (5**–**19)	12 (5**–**25)	0.225#
CRP POD 1–3 (mg/l) (*n* = 153)	127 (85**–**190)	176 (133**–**271)	< 0.001#
CRP POD 4–6 (mg/l) (*n* = 413)	81 (46**–**127)	151 (91**–**286)	< 0.001#
CRP POD 7–9 (mg/l) (*n* = 522)	53 (27**–**93)	128 (88**–**235)	< 0.001#
CRP POD 10–14 (mg/l) (*n* = 503)	38 (18**–**84)	119 (59**–**221)	< 0.001#
PCT POD 1 (ng/ml) (*n* = 33)	0.13 (0.08**–**0.25)	0.16 (0.09**–**1.38)	0.375#
PCT POD 1–3 (ng/ml) (*n* = 52)	0.46 (0.27**–**0.91)	1.18 (0.86**–**4.51)	< 0.001#
PCT POD 4–6 (ng/ml) (*n* = 43)	0.17 (0.13**–**0.34)	1.74 (0.42**–**4.39)	< 0.001#
PCT POD 7–9 (ng/ml) (*n* = 38)	0.10 (0.08**–**0.28)	1.52 (0.70**–**2.27)	< 0.001#
PCT POD 10–14 (ng/ml) (*n* = 36)	0.17 (0.10**–**1.19)	1.59 (0.89**–**5.05)	0.008#
Amylase POD 1 (U/l) (*n* = 676)	27 (15**–**49)	27 (19**–**52)	0.274#
Amylase POD 1–3 (U/l) (*n* = 746)	47 (13**–**190)	129 (42**–**379)	< 0.001#
Amylase POD 4–6 (U/l) (*n* = 612)	8 (4**–**20)	17 (9**–**39)	< 0.001#
Amylase POD 7–9 (U/l) (*n* = 299)	6 (3**–**16)	13 (7**–**22)	< 0.001#
Amylase POD 10–14 (U/l) (*n* = 380)	8 (4**–**19)	13 (7**–**27)	< 0.001#
Lipase POD 1 (U/l) (*n* = 474)	48 (28**–**114)	47 (28**–**104)	0.818#
Lipase POD 1–3 (U/l) (*n* = 198)	31 (10**–**194)	103 (40**–**277)	0.005#
Lipase POD 4–6 (U/l) (*n* = 247)	10 (6**–**20)	15 (9**–**32)	0.004#
Lipase POD 7–9 (U/l) (*n* = 267)	10 (7**–**21)	18 (10**–**30)	0.001#
Lipase POD 10–14 (U/l) (*n* = 253)	14 (8**–**27)	20 (11**–**41)	0.004#
GOT POD 1 (U/l) (*n* = 690)	33 (24**–**65)	30 (24**–**56)	0.247#
GOT POD 1–3 (U/l) (*n* = 408)	77 (49**–**123)	103 (59**–**183)	< 0.001#
GOT POD 4–6 (U/l) (*n* = 418)	38 (26**–**54)	40 (25**–**80)	0.133#
GOT POD 7–9 (U/l) (*n* = 398)	34 (24**–**51)	36 (26**–**61)	0.155#
GOT POD 10–14 (U/l) (*n* = 388)	34 (24**–**46)	36 (24**–**64)	0.112#
GPT POD 1 (U/l) (*n* = 506)	42 (22**–**108)	32 (21**–**84)	0.070#
GPT POD 1–3 (U/l) (*n* = 377)	70 (41**–**136)	78 (48**–**155)	0.096#
GPT POD 4–6 (U/l) (*n* = 392)	47 (29**–**77)	51 (27**–**100)	0.330#
GPT POD 7–9 (U/l) (*n* = 370)	44 (29**–**71)	46 (22**–**107)	0.911
GPT POD 10–14 (U/l) (*n* = 359)	39 (25**–**66)	36 (21**–**71)	0.647#
GGT POD 1 (U/l) (*n* = 526)	143 (38**–**439)	102 (33**–**341)	0.328#
GGT POD 1–3 (U/l) (*n* = 380)	76 (32**–**209)	84 (33**–**206)	0.616#
GGT POD 4–6 (U/l) (*n* = 405)	104 (51**–**213)	91 (49**–**202)	0.659#
GGT POD 7–9 (U/l) (*n* = 394)	127 (77**–**269)	167 (77**–**300)	0.365#
GGT POD 10–14 (U/l) (*n* = 384)	148 (78**–**277)	197 (108**–**382)	0.011#
AP POD 1 (U/l) (*n* = 698)	133 (85**–**301)	105 (74**–**234)	0.002#
AP POD 1–3 (U/l) (*n* = 325)	89 (60**–**178)	86 (51**–**164)	0.394#
AP POD 4–6 (U/l) (*n* = 374)	114 (74**–**200)	107 (75**–**167)	0.412#
AP POD 7–9 (U/l) (*n* = 375)	133 (89**–**210)	126 (86**–**174)	0.329#
AP POD 10–14 (U/l) (*n* = 368)	140 (97**–**231)	157 (107**–**255)	0.145#
Bilirubin POD 1 (mg/dl) (*n* = 708)	0.7 (0.4**–**2.2)	0.7 (0.4**–**2.0)	0.823#
Bilirubin POD 1–3 (mg/dl) (*n* = 751)	0.9 (0.6**–**1.8)	1.0 (0.6**–**2.0)	0.314#
Bilirubin POD 4–6 (mg/dl) (*n* = 658)	0.6 (0.3**–**1.2)	0.7 (0.4**–**1.8)	0.013#
Bilirubin POD 7–9 (mg/dl) (*n* = 503)	0.4 (0.3**–**0.9)	0.6 (0.4**–**1.2)	0.003#
Bilirubin POD 10–14 (mg/dl) (*n* = 443)	0.4 (0.3**–**0.9)	0.6 (0.4**–**1.3)	< 0.001#
Creatinine POD 1 (mg/dl) (*n* = 743)	0.8 (0.7**–**0.9)	0.8 (0.7**–**1.0)	0.001#
Creatinine POD 1–3 (mg/dl) (*n* = 758)	0.8 (0.7**–**1.0)	0.9 (0.7**–**1.2)	< 0.001#
Creatinine POD 4–6 (mg/dl) (*n* = 721)	0.7 (0.6**–**0.9)	0.8 (0.7**–**1.2)	< 0.001#
Creatinine POD 7–9 (mg/dl) (*n* = 666)	0.8 (0.7**–**0.9)	0.8 (0.7**–**1.1)	0.005#
Creatinine POD 10–14 (mg/dl) (*n* = 629)	0.8 (0.7**–**0.9)	0.8 (0.7**–**1.1)	0.014#
BUN POD 1 (mg/dl) (*n* = 535)	30 (24**–**38)	32 (26**–**42)	0.005#
BUN POD 1–3 (mg/dl) (*n* = 735)	35 (26**–**46)	42 (32**–**63)	< 0.001#
BUN POD 4–6 (mg/dl) (*n* = 626)	32 (24**–**46)	45 (30**–**68)	< 0.001#
BUN POD 7–9 (mg/dl) (*n* = 463)	27 (20**–**38)	39 (26**–**57)	< 0.001#
BUN POD 10–14 (mg/dl) (*n* = 420)	25 (19**–**33)	37 (24**–**54)	< 0.001#
Acute kidney injury POD 1–3			< 0.017*
No	531 (94.0%)	184 (88.9%)	
Yes	34 (6.0%)	23 (11.1%)	
Preoperative INR (*n* = 273)	0.99 (0.95**–**1.03)	0.99 (0.94**–**1.05)	0.502#
Preoperative PTT (s) (*n* = 271)	29 (28**–**32)	30 (28**–**32)	0.511#

*Pearson’s chi-square test

#Mann-Whitney *U*-test

**Fig. 1:**
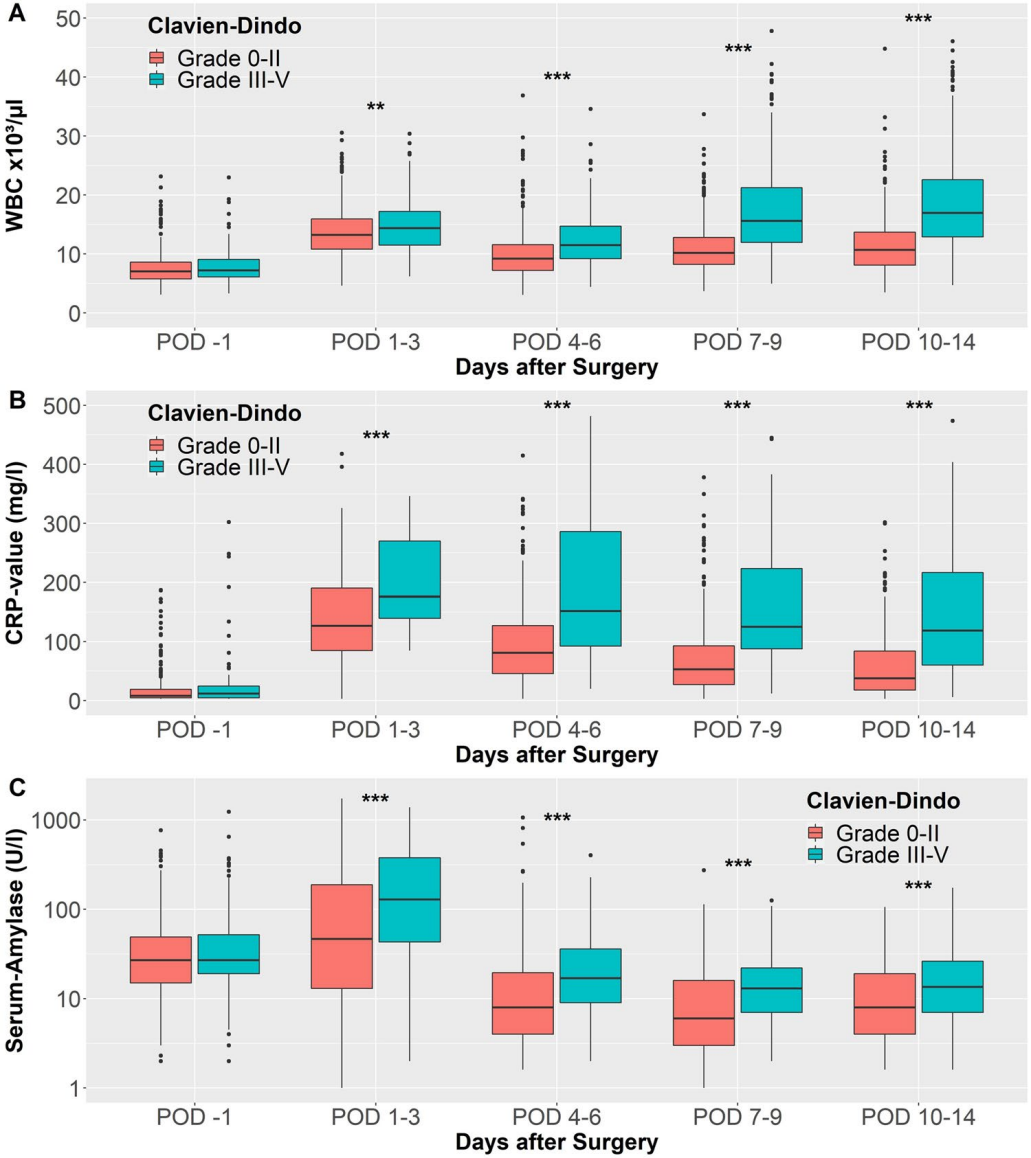
Laboratory course after pancreatic head resection of **A** white blood cell (WBC) count, **B** C-reactive protein (CRP), and **C** serum amylase (*p < 0.05, **p < 0.01; ***p < 0.001; Mann-Whitney *U*-test: Clavien-Dindo Grades 0–II vs. Clavien-Dindo Grades III–V)

### Preoperative parameters associated with increased risk for perioperative complications

Univariate analysis revealed several parameters associated with perioperative complications Clavien-Dindo Grade III or higher (Tables 1 and 2). In the preoperative setting, the patient’s age, the indication for surgery, CA19-9 value, ASA score, BMI, WBC POD 1, GPT POD 1, AP POD 1, creatinine POD 1, and BUN POD 1 were significantly associated with postoperative complications. The indications for surgery were associated with different risks for postoperative complications. Pancreatic surgery for IPMN, bile duct cancer, duodenal cancer, and “Other indication” (including MCNs, SCNs, schwannoma, GIST, and sarcoma) was associated with a higher risk for complications, while surgical resection of PDAC, chronic pancreatitis, pancreatic neuroendocrine tumors (pNET), and pancreatic metastases was associated with a reduced perioperative risk. Therefore, the indications were classified as indications with a high risk (IPMN, bile duct cancer, duodenal cancer, and “Other indications”) and indications with a low risk (PDAC, CP, pNET, and metastases) for postoperative complications. Multivariate analysis identified indication (risk-stratified), ASA score, BMI, WBC POD 1, and preoperative alkaline phosphatase as independent risk factors for postoperative complications (Table 3).

**Table 3:** Multivariate analysis of preoperative risk factors for postoperative complications ≥ Clavien-Dindo Grade III

Variable		Odds ratio	95%, CI	*p*-value
Indication (risk-stratified)	Low-risk indication	Reference	Reference	< 0.001
	High-risk indication	2.106	1.421–3.121	
ASA score	ASA 1–2	Reference	Reference	0.005
	ASA 3–4	1.690	1.176–2.431	
BMI	BMI < 30 Kg/m^2^	Reference	Reference	0.023
	BMI ≥ 30 Kg/m^2^	1.879	1.089–3.242	
WBC POD 1	< 9.5 × 10^3^	Reference	Reference	0.015
	≥ 9.5 × 10^3^	1.762	1.115–2.786	
Alkaline phosphatase POD 1	< 105 U/l	Reference	Reference	0.016
	≥ 105 U/l	0.634	0.437–0.920	

### Perioperative parameters associated with increased risk for perioperative complications

The associations of perioperative parameters with a clinically known outcome on the third postoperative day and postoperative complications were evaluated. Added to the preoperatively known parameters, operation time, the need for intraoperative blood transfusion, texture of pancreatic tissue, maximum drain amylase on POD 1–3, WBC POD 1–3, CRP POD 1–3, PCT POD 1–3, (serum) amylase POD 1–3, (serum) lipase POD 1–3, GOT POD 1–3, GPT POD 1–3, creatinine POD 1–3, BUN POD 1–3, and acute kidney injury on POD 1–3 are associated with postoperative complications. Multivariate analysis revealed that the indication (risk-stratified), ASA score, BMI, the need for intraoperative blood transfusion, prolonged operation time, WBC POD 1–3, (serum) amylase POD 1–3, and the preoperative alkaline phosphatase were independent prognostic factors of postoperative complications Clavien-Dindo Grade III or higher (Table 4). The texture of the pancreatic tissue is correlated with AP POD 1 and serum amylase POD 1–3, and therefore, these quantifiable variables without interobserver variability were used for multivariate analysis.

**Table 4:** Multivariate analysis of perioperative (preoperative to POD 3) risk factors for postoperative complications ≥ Clavien-Dindo Grade III

Variable		Odds ratio	95%, CI	*p*-value
Indication (risk-stratified)	Low-risk indication	Reference	Reference	0.005
	High-risk indication	1.845	1.204**–**2.827	
ASA score	ASA 1–2	Reference	Reference	0.003
	ASA 3–4	1.807	1.225**–**2.667	
BMI	BMI < 30 Kg/m^2^	Reference	Reference	0.043
	BMI ≥ 30 Kg/m^2^	1.813	0.019**–**3.224	
Intraoperative blood transfusion	No	Reference	Reference	0.001
	Yes	2.376	1.407**–**4.012	
Operation time	< 450 min	Reference	Reference	0.006
	≥ 450 min	1.746	1.173**–**2.600	
WBC POD 1–3	< 13.5 × 10^3^	Reference	Reference	0.015
	≥ 13.5 × 10^3^	1.614	1.096**–**2.378	
Serum amylase POD 1–3	< 54 U/l	Reference	Reference	< 0.001
	≥ 54 U/l	2.641	1.731**–**4.028	
Alkaline phosphatase POD 1	< 105 U/l	Reference	Reference	0.063
	≥ 105 U/l	0.679	0.451**–**1.021	

### Nomograms for prediction of postoperative complications

**Fig. 2:**
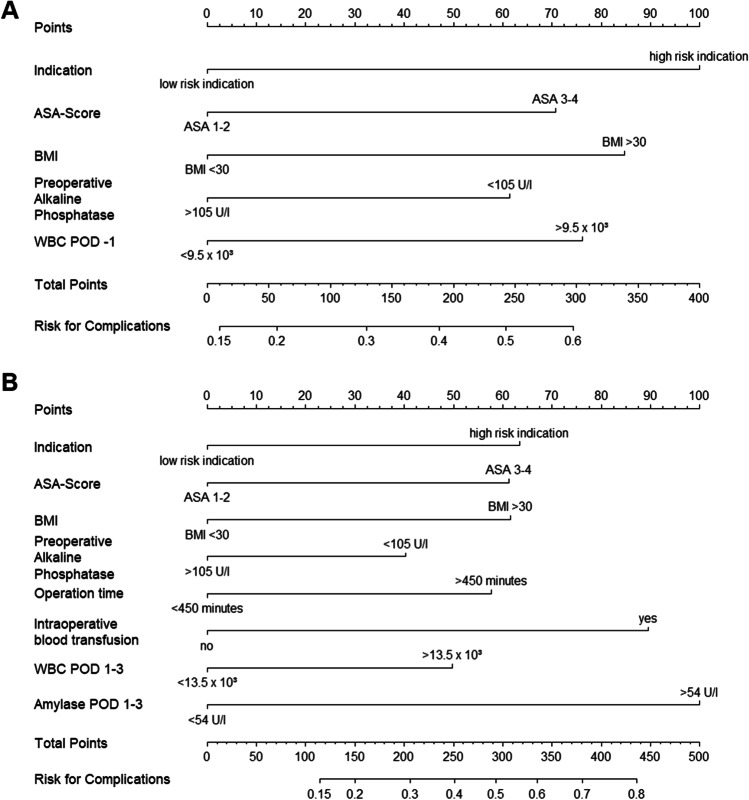
Nomograms for the estimation of the perioperative risk of severe (Clavien-Dindo Grades III–V) complications after pancreatic head resection**. A** Preoperative nomogram. **B** Postoperative nomogram (POD 3). (Low-risk indication: PDAC, CP, pNET, and metastases; High-risk indication: IPMN, bile duct cancer, duodenal cancer, other indications)

Based on the results of the multivariate analyses, two nomograms were developed (Fig. 2A and B, a step-by-step user instruction is available in the “Supplementary information” section Fig. S1). Calibration plots with bootstrap resampling were generated to correlate predicted and empirically observed probabilities of postoperative complications for internal validation. The calibration plots demonstrated high reliability in predicting postoperative complications for the preoperative and postoperative nomogram (Fig. S2A and B).

**Fig. 3:**
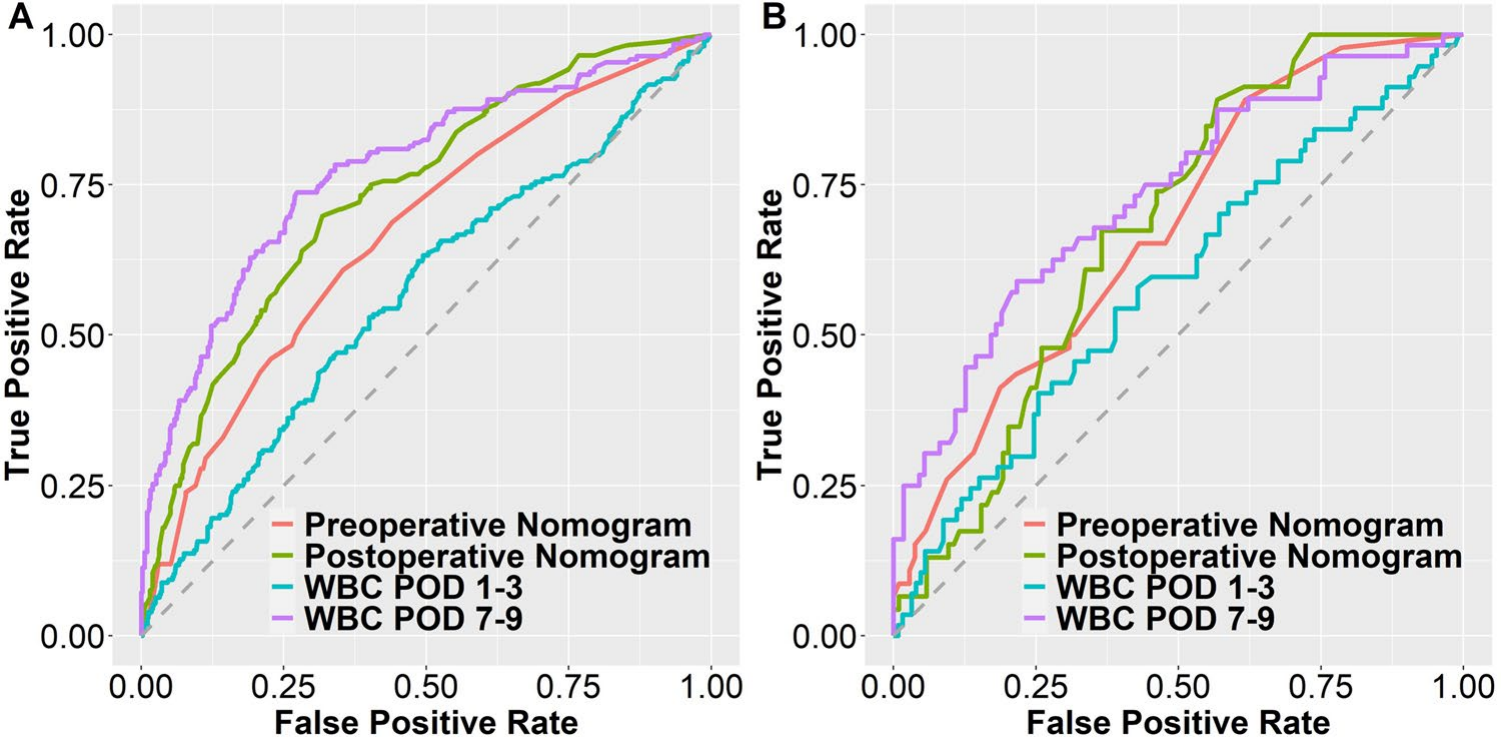
ROC analysis of the nomograms and WBC on POD 1–3 and 7–9 in the test and validation cohort**. A** ROC analysis of the nomograms compared to WBC on POD 1–3 and 7–9 in the test cohort. Test cohort ROC AUC values: preoperative nomogram: 0.673 (95%, CI: 0.626–0.721; *p* < 0.001), postoperative nomogram: 0.734 (95%, CI: 0.691–0.778; *p* < 0.001), WBC POD 1–3: 0.569 (95%, CI: 0.518–0.619 and *p*-value: 0.008), and WBC POD 7–9: 0.770 (95%, CI: 0.728–0.812; *p* < 0.001). **B** ROC analysis of the nomograms compared to WBC on POD 1–3 and 7–9 in the validation cohort. validation cohort ROC AUC values: preoperative nomogram: 0.676 (95%, CI: 0.586–0.766; *p* = 0.001), postoperative nomogram: 0.677 (95%, CI: 0.591–0.762; *p* = 0.001), WBC POD 1–3: 0.562 (95%, CI 0.465–0.659 and *p*-value: 0.212), and WBC POD 7–9: 0.706 (95%, CI: 0.618–0.793; *p*-value: 0.045).

External validation was performed by ROC analysis in the validation cohort. The AUC of the preoperative nomogram was 0.673 (95%, CI: 0.626–0.721; *p* < 0.001) in the test cohort and 0.676 (95%, CI: 0.586–0.766; *p* = 0.001) in the validation cohort. The AUC of the postoperative (POD 1–3) nomogram was 0.734 (95%, CI: 0.691–0.778; *p* < 0.001) and 0.677 (95%, CI: 0.591–0.762; *p* = 0.001) in the validation cohort. WBC showed an AUC of 0.552 on POD 1 (95%, CI: 0.502–0.603; *p* = 0.041), 0.569 on POD 1–3 (95%, CI: 0.518–0.619, *p* = 0.008), 0.670 on POD 4–6 (95%, CI: 0.623–0.716; *p* < 0.001), and 0.770 on POD 7–9 (95%, CI: 0.728–0.812; *p* < 0.001). The predictive value of the postoperative nomogram was therefore comparably accurate on POD 3 as the diagnostic value of WBC on POD 7–9. In the validation cohort, the AUC of the WBC was 0.562 on POD 1–3 (95%, CI 0.465–0.659, *p* = 0.212) and 0.706 (95%, CI: 0.618–0.793; *p* = 0.045) on POD 7–9 (Fig. 3A and B).

Although not designed for this specific task, prediction of Clavien-Dindo Grades IV and V also was possible with the nomograms (Supplementary information Table S1 and Supplementary information Fig. S3). We compared our nomogram with the FRS—which is calculated postoperatively—in a cohort of 57 patients from our validation cohort, for which all data needed for the calculation of the FRS were available. AUC for prediction of complications Clavien-Dindo Grade III or higher was 0.672 (*p* = 0.030) for the FRS, 0.665 (*p* = 0.033) for the preoperative nomogram, and 0.674 (*p* = 0.020) for the postoperative nomogram. Therefore, both postoperative scores were almost equally efficient in the prediction of postoperative complications and the preoperative nomogram only slightly less accurate. Combination of the FRS and our nomograms by simple addition of normalized score values showed that the FRS and the preoperative nomogram improved the predictive accuracy to AUC 0.696 (*p* = 0.012) and the FRS and the postoperative nomogram to AUC 0.682 (*p* = 0.017).

### Different patterns of postoperative complications due to preoperative risk factors

When comparing the risk for certain postoperative complications, different patterns of complications due to the different preoperative risk factors can be observed. While patients with high-risk indications or a low preoperative alkaline phosphatase have a higher risk for POPF and subsequently a higher risk for postoperative sepsis, intensive care-associated complications such as pneumonia, thromboembolism, and acute renal failure are more likely to occur in patients with a higher ASA score. The first diagnostic procedure to diagnose postoperative complications was performed on POD 8 (4–11.5) (computed tomography: *n* = 212 and endoscopy: *n* = 54) and the first intervention for treatment of postoperative complications was also performed on POD 8 (4–12). The first postoperative interventions are summarized in Table S6. Elevated WBC (≥ 12.5 × 10^3^/μl) and CRP (≥ 85 mg/l) levels in the later postoperative course POD 7–9 implicate a higher rate of operative revision procedures and less interventional procedures, indicating a higher severity of the clinical condition of patients with these laboratory findings (Supplementary information Tables S5, S6, and S7).

## Discussion

In this study, we were able to develop two nomograms for prediction of perioperative morbidity and mortality in the preoperative and perioperative setting. The nomograms demonstrated both high reliability in internal validation by bootstrap resampling as well as a good fitness in external validation with the completely independent validation cohort of this study.

The identified risk factors for the preoperative nomogram are the indication for surgery, the ASA score, the BMI, the preoperative WBC, and the preoperative alkaline phosphatase. The correlation between perioperative risks and the different indications for pancreatic head resection is well known. While surgical resection of PDAC and chronic pancreatitis is known to have less perioperative risk mainly due to fibrous pancreatic tissue and a lower risk of POPF, patients with IPMN, MCN, distal bile duct cancer, and duodenal cancer are at increased risk for POPF and postoperative complications [3, 26–28]. In our cohort, pNETs were surprisingly not associated with an increased risk of perioperative complications and therefore had to be stratified as a “low-risk indication”. Other authors describe an increased risk in pancreatic surgery for pNET compared to other indications [29, 30]. The ASA score is a well-established parameter for general evaluation of perioperative risk in surgery and for pancreatic surgery in particular as well [31, 32] and an increased body mass index is also a well-established risk factor for postoperative surgical and medical complications [29, 33, 34]. Preoperative WBC and especially postoperative WBC are known factors for the prediction of postoperative complications in general surgery [35, 36] and for pancreatic resections in particular [37, 38].

A very interesting observation was made in our data when analyzing the preoperative alkaline phosphatase levels. Patients with increased preoperative alkaline phosphatase levels ≥ 105 U/l were at decreased risk for postoperative complications and had less POPF. This association has not been described before and warrants further research. Alkaline phosphatase is known to be elevated in patients with malignant tumors of the pancreas [39], with IPMNs with progression during observation [40], and correlates with CA19-9 values [41]. A possible explanation of this observation is the known impact of pancreatic duct occlusion on alkaline phosphatase levels and a possible activation of fibroblasts in the pancreas due to this mechanism [42].

The postoperative nomogram consists of the preoperatively known parameters listed above without the preoperative WBC, and is enhanced with parameters with a known outcome on POD 3. The first new variable in the postoperative nomogram is a prolonged operation time. Earlier studies showed that operation time is an independent risk factor for an unfavorable postoperative outcome and should be considered as a relevant parameter in risk-adjustment processes and as a possible area of quality improvement on the individual and system level [43, 44]. Perioperative blood transfusions are known to be associated with an increased risk for postoperative complications [44, 45] and prudent patient-blood management with adequate transfusion triggers is essential for prevention of unnecessary transfusions [46]. Accordingly, the need for intraoperative blood transfusion is also a relevant prognostic factor of postoperative complications with a high impact on the overall nomogram score.

Furthermore, the serum amylase POD 1–3 levels are a strong predictor of postoperative complications in our cohort. Early postoperative serum amylase, as a marker for postoperative pancreatitis, is known to predict POPF [47, 48]. As POPF is one of the main reasons for postoperative complications following pancreatic head resection, an odds ratio of 2.64 of patients with elevated serum amylase levels and the highest impact on the overall score of the postoperative nomogram can be observed in our cohort. Our nomograms for prediction of postoperative complications therefore have some similarities, but also differences to scores predicting POPF such as the ASA score, BMI, intraoperative blood transfusions, and the indication for pancreatic surgery are part of the fistula risk score (FRS) [26] or a recently-published nomogram [12]. Other scoring systems to predict POPF exist. A systematic review summarized the existing scoring systems. Interestingly, neither preoperative alkaline phosphatase nor postoperative serum amylase or serum lipase is used as a specific risk factor in prediction of pancreatic fistula in any relevant fistula risk score [49], although postoperative serum amylase and postoperative hyperlipasemia accurately predict the risk for POPF [47, 48].

Maximum drain-amylase levels on POD 1–3 [50, 51], advanced age [52], pre-existing elevated creatinine [53], and postoperative acute renal failure [54] are known risk factors for postoperative complications, but failed to improve the predictive value of the logistic regression models in our cohort and were therefore excluded during multivariate analysis.

Limitations of this study are, of course, the retrospective design of the study with the resultant missing laboratory data during the postoperative course and missing preoperative variables. Low protein and albumin levels for example are known predictors of postoperative complications after general [55] and pancreatic surgery [56]. These parameters were not routinely measured in our perioperative work-up of patients and therefore were not included in the nomogram development. During a prospective validation study of the nomograms, these parameters should additionally be obtained routinely for possible refinement of the predictive value of the risk scores. Additionally, radiological factors and probably the assessment of the pancreatic gland texture with the help of radiomics can further refine the risk assessment in the future.

Nevertheless, the established nomograms demonstrated good internal and external validity and improve the pre- and perioperative assessment of patients scheduled for pancreatic head resection. In our opinion, there are two main clinical applications for the preoperative nomogram. First of all, surgeons can assess the individual perioperative risk for a patient. For example, when combining our nomogram with risk scores for the prediction of malignancy in cystic tumors of the pancreas [57, 58], informed consideration of surgical risk and risk of malignancy can help to decide whether a surgical resection should be performed in patients at a higher risk for surgical complications. Secondly, the nomogram can aid in the decision-making process with a patient. With the help of this nomogram, a patient can better understand his individual risk and a decision for or against surgery can be made with better informed consent. The positive effect of a preoperative weight loss can also be demonstrated to obese patients in whom the resection is not urgently required, for example, in patients with prophylactic resection of IPMN with worrisome features. The postoperative nomogram can help to evaluate the individual risk of complications after the operation and might help to increase the awareness of the treatment team towards a high-risk patient and support early detection and early treatment of complications, thereby preventing the sequelae of delayed complication management.

In conclusion, we developed and validated two nomograms for prediction of severe perioperative complications in the preoperative and perioperative setting after pancreatic head resection. We could demonstrate that both nomograms also predict higher grade complications Clavien-Dindo Grades IV–V and V, respectively, making both nomograms a solid new tool for estimation of perioperative risk for an individual patient

## Supplementary Information

ESM 1 (DOCX 7829 kb)
