# Supplementary material for: Perioperative risk of pancreatic head resection—nomogram-based prediction of severe postoperative complications as a decisional aid for clinical practice
Source: Langenbecks Arch Surg. 2022 Mar 23;407(5):1935–47. doi: 10.1007/s00423-021-02426-z (PMC9399026; doi:10.1007/s00423-021-02426-z)
Supplement: Supplementary file 1 — (DOCX 7829 kb) [file 423_2021_2426_MOESM1_ESM.docx]

**Supplementary Information:**

**Perioperative Risk of Pancreatic Head Resection – Nomogram-based prediction of severe postoperative complications as a decisional aid for clinical practice**

**J. Hipp, L. Rist, S. Chikhladze, D.A. Ruess, S. Fichtner-Feigl and U.A. Wittel**

**Fig. S1: How to use the nomograms?** Example: A fictitious patient with an IPMN (high risk indication), ASA 3-classification, a BMI <30 kg/m² and a preoperative alkaline phosphatase of > 105 U/l underwent pancreatic head resection. The operation had a duration of less than 450 minutes and no intraoperative blood transfusions were needed. The WBC peaked on POD 2 with >13.5x10³/µl and the Serum-Amylase peaked on POD 1 with >54 U/l. The nomogram-score in the postoperative nomogram is therefore 63+61+50+100 = 274. This relates to a risk of ~45% for complications Clavien-Dindo grade III or higher during the postoperative course.


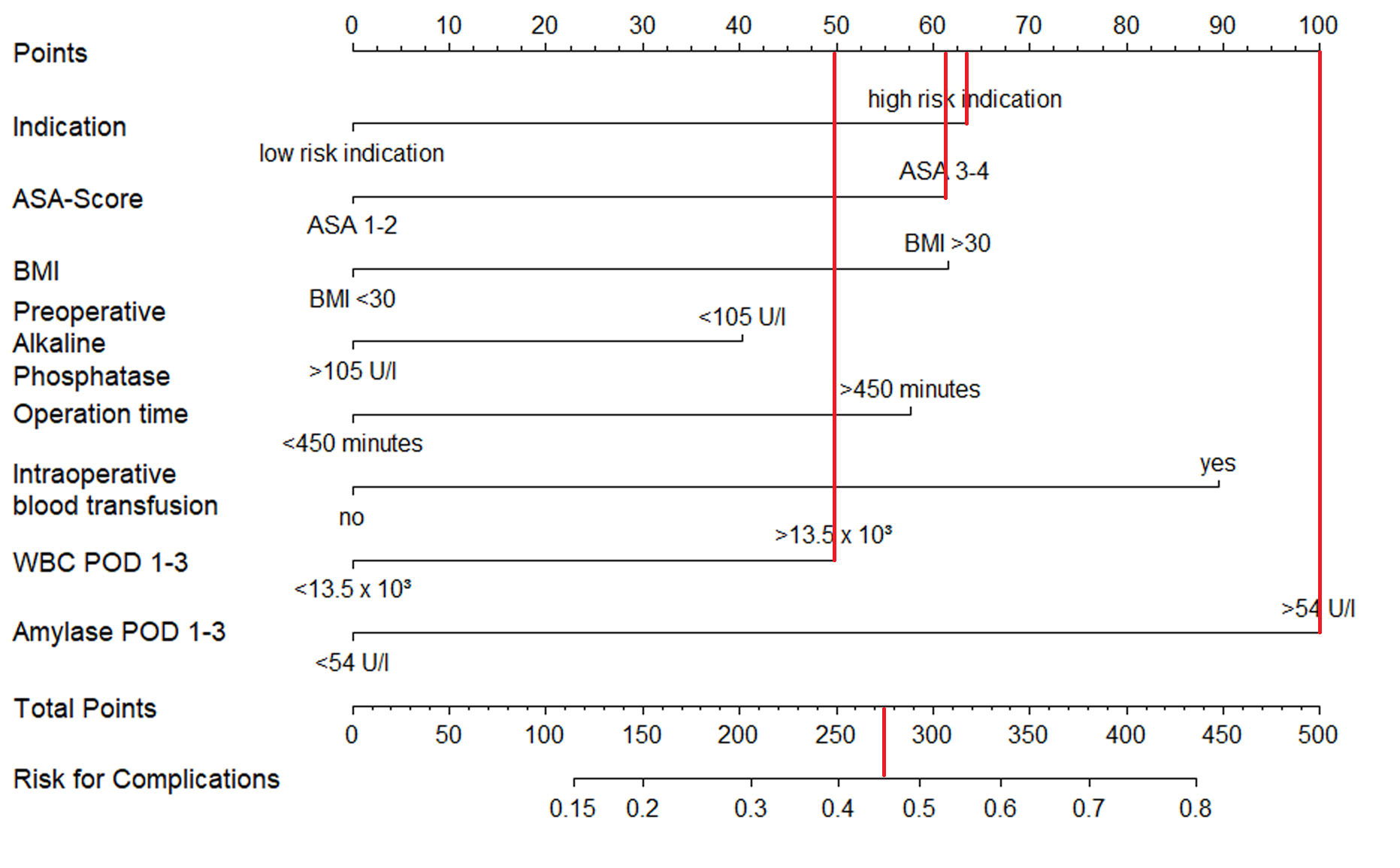


**Fig. S2: Calibration plots of the nomograms**

A) Calibration plot of the preoperative Nomogram

B) Calibration plot of the postoperative Nomogram


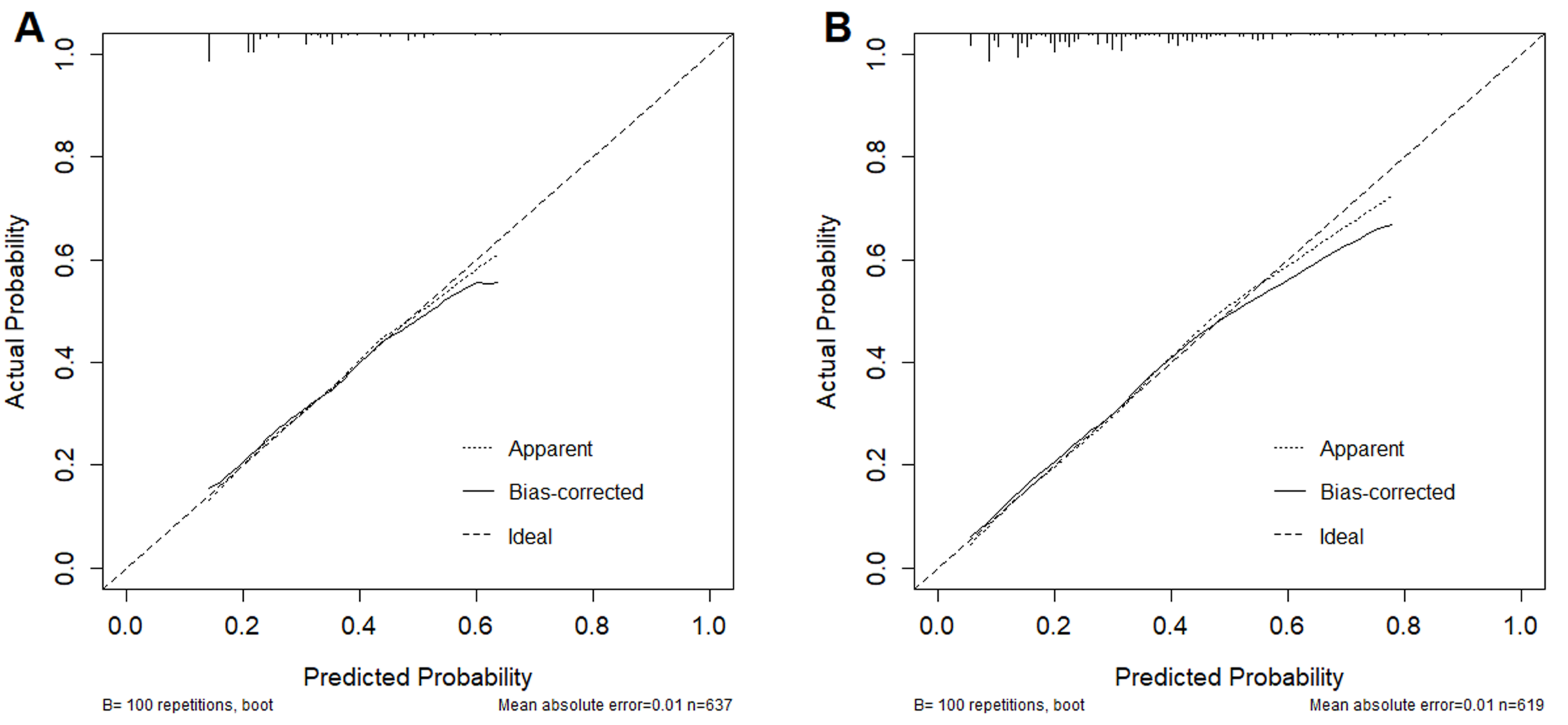


**Fig. S3: ROC-analyses of preoperative and postoperative nomogram for the prediction of complications Clavien-Dindo grade IV-V and postoperative death (Grade V)**

A) ROC-analysis for the prediction of complications grade IV-V in the test cohort. Preoperative Nomogram: AUC 0.689, 95%-CI: 0.606-0.772; p<0.001; Postoperative Nomogram: AUC 0.712, 95%-CI: 0.636-0.789; p<0.001.

B) ROC-analysis for the prediction of complications grade IV-V in the validation cohort. Preoperative Nomogram: AUC 0.636 (95%-CI: 0.496-0.776; p=0.095); Postoperative Nomogram: AUC 0.612 (95%-CI: 0.481-0.742; p=0.170).

C) ROC-analysis for the prediction of complications grade V in the test cohort. Preoperative Nomogram: AUC 0.691 (95%-CI: 0.588-0.795; p=0.001); Postoperative Nomogram: AUC 0.691 (95%-CI: 0.586-0.797; p=0.001).

D) ROC-analysis for the prediction of complications grade V in the validation cohort. Preoperative Nomogram: AUC 0.694 (95%-CI: 0.334-1.00; p=0.185); Postoperative Nomogram: AUC 0.742 (95%-CI: 0.483-1.00; p=0.099).





**Table S1: Clinical and laboratory data of validation cohort**

|  | **Clavien-Dindo Grade 0-II**  **(n=126)** | **Clavien-Dindo ≥Grade III**  **(n=58)** | **p-value** |
| --- | --- | --- | --- |
| **Age (years) (n=184)** | 66.5 (57-73) | 70 (60-76) | 0.091# |
| **Gender** | | | 0.801* |
| Male | 67 (53.2%) | 32 (55%) |  |
| Female | 59 (46.8%) | 26 (45%) |  |
| **Indication (risk-stratified)** | | | 0.001* |
| Low risk indication  (PDAC, CP, pNET, Metastases) | 102 (81.0%) | 33 (57%) |  |
| High risk indication  (IPMN, bile duct cancer, duodenal cancer, Other indications) | 24 (19.0%) | 25 (43%) |  |
| **ASA-Score** | | | 0.160* |
| ASA 1-2 | 66 (56.9%) | 24 (45%) |  |
| ASA 3-4 | 50 (43.1%) | 29 (55%) |  |
| **BMI** | | | 0.056* |
| BMI <30 Kg/m² | 113 (89.7%) | 46 (79%) |  |
| BMI ≥ 30Kg/m² | 13 (10.3%) | 12 (21%) |  |
| **Operation** | | | 0.076* |
| Whipple-Procedure | 11 (8.7%) | 8 (14%) |  |
| PPPD | 100 (79.4%) | 37 (64%) |  |
| Lap. ass. PPPD | 15 (11.9%) | 13 (22%) |  |
| **Operation time (min.) (n=183)** | 423 (357-481) | 402 (332-476) | 0.248# |
| **Intraoperative blood loss (ml) (n=106)** | 600 (300-1 000) | 600 (325-1 000) | 0.747# |
| **Intraoperative blood transfusion** | | | 0.880* |
| No | 99 (78.6%) | 45 (78%) |  |
| Yes | 27 (21.4%) | 13 (22%) |  |
| **POPF ISGPS** | | | <0.001* |
| Grade B | 15 (11.9%) | 15 (26%) |  |
| Grade C | 0 (0%) | 15 (26%) |  |
| **DGE ISGPS** | | | <0.001* |
| Grade B | 13 (10.3%) | 10 (17%) |  |
| Grade C | 3 (2.4%) | 22 (28%) |  |
| **PPH ISGPS** | | | <0.001* |
| Grade B | 2 (1.6%) | 10 (17%) |  |
| Grade C | 0 (0%) | 8 (14%) |  |
| **Clavien-Dindo-Classification** | | | <0.001* |
| Grade 0-II | 126 (100%) | 0 (0%) |  |
| Grade III | 0 (0%) | 43 (74%) |  |
| Grade IV | 0 (0%) | 11 (19%) |  |
| Grade V | 0 (0%) | 4 (7%) |  |
| **Comprehensive Complication-Index (CCI) (n=184)** | 20.9 (0-24.2) | 43.65 (34.33-64.58) | <0.001# |
| **Acute kidney injury** | | | 0.013* |
| No | 120 (95.2%) | 49 (85%) |  |
| Yes | 6 (4.8%) | 9 (15%) |  |
| **WBC POD -1 (x10³/µl) (n=177)** | 7.00 (5.90-8.73) | 7.40 (6.28-9.43) | 0.372# |
| **WBC POD 1-3 (x10³/µl) (n=183)** | 13.55 (10.35-16.32) | 14.39 (11.92-18.45) | 0.071# |
| **CRP POD -1 (mg/l) (n=77)** | 9 (5-21) | 10 (6-13) | 0.700# |
| **CRP POD 1-3 (mg/l) (n=33)** | 169 (94-213) | 200 (100-287) | 0.358# |
| **Amylase POD -1 (U/l) (n=159)** | 25 (15-41) | 27 (14-41) | 0.919# |
| **Amylase POD 1-3 (U/l) (n=180)** | 47 (14-206) | 220 (42-474) | <0.001# |
| **AP POD -1 (U/l) (n=162)** | 138 (88-322) | 121 (75-201) | 0.111# |
| **Postoperative Hospital stay (days) (n=182)** | 15 (12-18) | 30 (22-42) | <0.001# |

*Pearson’s chi square-test

#Mann-Whitney-U-test

**Table S2: Kruskal-Wallis-test and Mann-Whitney-U-posthoc-test with Bonferroni-correction of association between POPF and preoperative Alkaline Phosphatase**

| **Alkaline Phosphatase** |  | **No POPF** | **POPF biochemical leak** |
| --- | --- | --- | --- |
|  | **POPF biochemical leak** | <0.001 |  |
|  | **POPF Grade B/C** | <0.001 | 0.976 |

**Table S3: Preoperative Nomogram – Points and cut-off values**

| **Variable** | **Points** |
| --- | --- |
| **Indication** |  |
| low risk indication | 0 |
| high risk indication | 100 |
| **ASA-Score Points** |  |
| ASA 1-2 | 0 |
| ASA 3-4 | 71 |
| **BMI Points** |  |
| < 30 Kg/m² | 0 |
| ≥ 30 Kg/m² | 85 |
| **Preoperative Alkaline Phosphatase Points** |  |
| <105 U/l | 61 |
| ≥105 U/l | 0 |
| **WBC POD -1 Points** |  |
| <9.5 x10³/µl | 0 |
| ≥9.5 x10³/µl | 76 |
| **Total Points** | **Risk for Complications**  **Grade III-V** |
| 10 | 0.15 |
| 57 | 0.2 |
| 129 | 0.3 |
| 188 | 0.4 |
| 243 | 0.5 |
| 297 | 0.6 |

**Table S4: Postoperative Nomogram – Points and cut-off values**

| **Variable** | **Points** |
| --- | --- |
| **Indication** |  |
| low risk indication | 0 |
| high risk indication | 63 |
| **ASA-Score Points** |  |
| ASA 1-2 | 0 |
| ASA 3-4 | 61 |
| **BMI Points** |  |
| < 30 Kg/m² | 0 |
| ≥ 30 Kg/m² | 62 |
| **Preoperative Alkaline Phosphatase Points** |  |
| <105 U/l | 40 |
| ≥105 U/l | 0 |
| **Operation time Points** |  |
| <450 minutes | 0 |
| ≥450 minutes | 58 |
| **Intraoperative blood transfusion** |  |
| No | 0 |
| Yes | 90 |
| **WBC POD 1-3 Points** |  |
| <13.5 x10³/µl | 0 |
| ≥13.5 x10³/µl | 50 |
| **Serum-Amylase POD 1-3 Points** |  |
| <54 U/l | 0 |
| ≥54 U/l | 100 |
| **Total Points** | **Risk for Complications**  **Grade III-V** |
| 114 | 0.15 |
| 150 | 0.2 |
| 206 | 0.3 |
| 251 | 0.4 |
| 293 | 0.5 |
| 335 | 0.6 |
| 381 | 0.7 |
| 436 | 0.8 |

**Table S5: Postoperative Complications correlated to preoperative risk factors (all patients; n=956)**

| **Complication** | | **Indication (risk stratified)** | | **ASA-Score** | | **BMI** | | **Alkaline Phosphatase** | | **WBC POD -1** | |
| --- | --- | --- | --- | --- | --- | --- | --- | --- | --- | --- | --- |
|  |  | Low risk | High risk | ASA 1-2 | ASA 3-4 | < 30 Kg/m² | ≥ 30 Kg/m² | <105 U/l | ≥105 U/l | <9.5 x10³/µl | ≥9.5 x10³/µl |
| **POPF Grade B/C** | No. | 123 | 89 | 109 | 83 | 177 | 36 | 102 | 95 | 167 | 42 |
|  | % | 17.4 | 35.9^§^ | 22.0 | 22.6 | 20.8 | 34.3^§^ | 29.1 | 18.6^§^ | 21.7 | 26.6 |
| **PPH Grade B/C** | No. | 57 | 40 | 41 | 50 | 77 | 20 | 36 | 46 | 74 | 13 |
|  | % | 8.1 | 16.2^§^ | 8.2 | 13.7^§^ | 9 | 19.3^§^ | 10.3 | 9.1 | 9.6 | 8.3 |
| **DGE Grade B/C** | No. | 148 | 61 | 93 | 90 | 177 | 32 | 63 | 89 | 142 | 26 |
|  | % | 21.0 | 24.7 | 18.8 | 27.4^§^ | 20.9 | 30.8 | 18.1 | 17.7 | 18.6 | 16.6 |
| **Postop. Pneumonia** | No. | 30 | 23 | 21 | 27 | 45 | 8 | 27 | 24 | 40 | 12 |
|  | % | 4.3 | 9.3^§^ | 4.2 | 7.4^§^ | 5.3 | 7.7 | 7.8 | 4.7 | 5.2 | 7.6 |
| **Postoperative Sepsis** | No. | 24 | 19 | 21 | 20 | 35 | 8 | 22 | 17 | 33 | 8 |
|  | % | 3.4 | 7.7^§^ | 4.2 | 5.5 | 4.1 | 7.7 | 6.3 | 3.3 | 4.3 | 5.1 |
| **Acute Renal Failure** | No. | 50 | 22 | 30 | 39 | 57 | 15 | 23 | 43 | 64 | 8 |
|  | % | 7.1 | 8.9 | 6.0 | 10.6^§^ | 6.7 | 14.3^§^ | 6.6 | 8.4 | 8.3 | 5.1 |
| **Thromboembolism** | No. | 18 | 12 | 8 | 20 | 26 | 4 | 16 | 13 | 25 | 5 |
|  | % | 2.6 | 4.8 | 1.6 | 5.5^§^ | 3.1 | 3.8 | 4.6 | 2.6 | 3.3 | 3.2 |

^§^ p<0.05; Pearson’s chi square test

**Table S6: First postoperative Interventions due to complications correlated to different laboratory parameters (all patients with Clavien-Dindo grade III-V)**

|  | | **WBC POD 7-9** | | **CRP POD 7-9** | | **Amylase POD 7-9** | | **Bilirubin POD 7-9** | |
| --- | --- | --- | --- | --- | --- | --- | --- | --- | --- |
|  |  | <12.5 x 10³/µl* | ≥12.5 x 10³/µl* | <85 mg/l* | ≥85 mg/l* | <7 U/l* | ≥7 U/l* | <1.4 mg/dl* | ≥1.4 mg/dl* |
| **Drainage (CT- or US-guided)** | No. | 17 | 45 | 12 | 33 | 7 | 24 | 32 | 11 |
|  | % | 23.9 | 27.9 | 27.9 | 27.5 | 20.0 | 25.0 | 21.9 | 28.2 |
| **Surgical revision** | No. | 25 | 86 | 15 | 57 | 16 | 46 | 72 | 21 |
|  | % | 35.2 | 51.8 | 34.9 | 47.5 | 45.7 | 47.9 | 49.3 | 53.8 |
| **Endoscopic Intervention/PTCD** | No. | 13 | 7 | 7 | 5 | 3 | 7 | 10 | 2 |
|  | % | 18.3 | 4.2 | 16.3 | 4.2 | 8.6 | 7.3 | 6.8 | 5.1 |
| **Angiography** | No. | 4 | 9 | 3 | 6 | 3 | 5 | 8 | 3 |
|  | % | 5.6 | 5.4 | 7.0 | 5.0 | 8.6 | 5.2 | 5.5 | 7.7 |
| **Only intensive care treatment** | No. | 7 | 10 | 3 | 10 | 5 | 6 | 12 | 2 |
|  | % | 9.9 | 6.0 | 7.0 | 8.3 | 14.3 | 6.3 | 8.2 | 5.1 |

*Ideal cutoff-values were determined with ROC-analysis and Youden-Index.

**Table S7: Severity of postoperative complications for preoperative risk factors (all patients; n=956)**

|  | **Indication (risk stratified)** | | **ASA-Score** | | **BMI** | | **Alkaline Phosphatase** | | **WBC POD -1** | |
| --- | --- | --- | --- | --- | --- | --- | --- | --- | --- | --- |
| **Clavien-Dindo Classification** | ***Low risk*** | ***High risk*** | ***ASA 1-2*** | ***ASA 3-4*** | ***< 30 Kg/m²*** | ***≥ 30 Kg/m²*** | ***<105 U/l*** | ***≥105 U/l*** | **<9.5 x10³** | **≥9.5 x10³** |
| **Grade 0-II** | 546 (77.2) | 145 (58.5) | 379 (76.4) | 240 (65.2) | 628 (73.8) | 63 (60.0) | 234 (66.9) | 390 (76.5) | 568 (73.8) | 101 (63.9) |
| **Grade III-V** | 161 (22.8) | 103 (41.5) | 117 (23.6) | 128 (34.8) | 223 (26.2) | 42 (40.0) | 116 (33.1) | 120 (23.5) | 202 (26.2) | 57 (36.1) |
| **p-value** | <0.001 | | <0.001 | | 0.003 | | 0.002 | | 0.012 | |
